# Assessment of the Clinical Efficacy and Safety of Azithromycin in Patients With Moderate to Severe Upper Respiratory Tract Infections (URTIs): Insights From an Indian Real-World Study

**DOI:** 10.7759/cureus.89403

**Published:** 2025-08-05

**Authors:** Harihara Murthy, Pawan Singhal, Carlton Pereira, Paritosh Pandey, Ranjan Kumar Jha, Bimarjeet Pradhan, Onkar C Swami, Dinesh R Patil, Darshan D Rana

**Affiliations:** 1 Department of Ear, Nose, and Throat (ENT), Apollo Spectra Hospitals, Bangalore, IND; 2 Department of Ear, Nose, and Throat (ENT), Lipi Clinic, Jaipur, IND; 3 Department of Ear, Nose, and Throat (ENT), Bosco ENT Centre, Mumbai, IND; 4 Department of Ear, Nose, and Throat (ENT), Manas ENT and Allergy Institute, Lucknow, IND; 5 Department of Ear, Nose, and Throat (ENT), Santevita Hospital, Ranchi, IND; 6 Department of Ear, Nose, and Throat (ENT), Apex Hospital, Jamshedpur, IND; 7 Department of Medical Services, Alembic Pharmaceuticals Limited, Mumbai, IND

**Keywords:** macrolides, pharyngitis, sore throat, tonsillitis, tonsillopharyngitis

## Abstract

Introduction

Upper respiratory tract infections (URTIs) are commonly encountered in primary care. Azithromycin has emerged as a preferred therapy for URTIs due to its once-daily dosing, low resistance risk, and favorable gastrointestinal tolerability. This study evaluated the real-world effectiveness and safety of azithromycin (500 mg/day) in moderate to severe acute URTIs.

Materials and methods

This subgroup analysis of a retrospective, multicenter, observational study analyzed data from 184 ENT centers in India over 12 weeks. Medical records of adult patients with moderate to severe URTIs treated with azithromycin 500 mg daily for five days were included. Key clinical signs (pharyngeal and tonsillar erythema, exudates) and symptoms (sore throat, fever, daily activity interference) were assessed on a 3-point scale. Treatment effectiveness was evaluated through changes in cumulative symptom scores and Clinical Global Impression scores on day 5. Data were analyzed using paired t-tests and McNemar-Bowker tests, with p<0.05 indicating significance. Ethical approval was obtained, and all data collection adhered to Good Clinical Practice standards.

Results

Data from 427 patients with moderate to severe URTIs were analyzed. By day 5, significant improvements were observed across all clinical parameters. Proportion of patients with moderate pharyngeal erythema (40.7%) and severe cases (59.3%) reduced significantly to 16.6% and 0.2%, respectively, and severe tonsillar erythema (63%) reduced from baseline to no cases with severe tonsillar erythema at day 5. Tonsillar exudates reduced from 52.9% to 7.6% for moderate cases and from 47.1% to 0.2% for severe cases. Sore throat severity dropped from 73.5% to only 0.2% reporting difficulty swallowing. Fever resolved in 96.7% of patients, and work absenteeism reduced from 71.2% to 0.9%. Clinical Global Impression scores indicated improvement in 90% of patients. Adverse events were reported in only 2.1% of cases.

Conclusion

Azithromycin demonstrated significant clinical effectiveness in managing moderate to severe URTIs, with marked improvements in key symptoms and signs.

## Introduction

Respiratory infections continue to pose a significant public health challenge worldwide. Both acute and chronic respiratory diseases are major contributors to morbidity and mortality, particularly in developing countries, and consequent economic costs [1]. Upper respiratory tract infections (URTIs), such as pharyngitis, tonsillitis, nasopharyngitis, rhinitis, and laryngitis, are among the most frequently encountered conditions in primary healthcare settings. According to the Global Burden of Disease Study 2019, India reported an incidence of 2.70 billion URTIs in 2019. The age-standardized mortality rate (ASMR) for URTIs in India is 0.066 per 100,000 [2].

In real-world clinical practice, the diagnosis of bacterial URTIs is primarily based on clinical judgment, relying on the presence of specific signs and symptoms indicative of a bacterial origin. While most URTIs are viral, some cases involve primary bacterial infections or superinfections that necessitate antibiotic treatment. Common bacterial culprits include group A beta-hemolytic *Streptococcus* (GABHS), *Streptococcus pneumoniae*, *Haemophilus influenzae*, and *Moraxella catarrhalis* [3]. Acute tonsillopharyngitis affects the palatine tonsils and pharynx, presenting with symptoms such as erythema, exudation, or ulceration. Patients with moderate to severe cases require immediate and effective antibiotic treatment [4].

In cases presenting with URTIs, antibiotics are typically considered only when clinical evaluation suggests a likely bacterial etiology. In particular, guidelines recommend macrolides and penicillins for treating URTIs attributed to group A *Streptococcus*. Emphasizing the rational use of antibiotics is essential to minimize the risk of antimicrobial resistance, reduce unnecessary drug exposure, and promote effective, evidence-based care. Due to significant compliance barriers with other antibiotics, azithromycin has become a popular choice for treating URTIs. Its once-daily dosing for five days, low risk of resistance, and lower risk of gastrointestinal side effects make it convenient for patients [5,6]. Azithromycin, with its azalide structure, is stable in the acidic environment of the stomach and effective against gram-negative and atypical pathogens such as *Mycoplasma pneumoniae* and *Chlamydophila pneumoniae*, which can also cause acute respiratory infections, including pharyngitis [6,7].

Currently, there is limited recent data from India on the effectiveness and safety of azithromycin in treating moderate to severe acute URTIs, particularly after the COVID-19 pandemic. Most previous studies evaluating azithromycin's role in URTIs have focused on mild to moderate cases, with limited real-world data on moderate to severe presentations in India. Severe cases due to delayed bacterial eradication could lead to complications such as peritonsillar abscess or rheumatic fever, which remain concerns in India. A targeted analysis of moderate to severe cases will help determine if azithromycin remains a viable first-line or adjunct therapy in this subset. This study aims to evaluate the effectiveness and safety of azithromycin 500 mg/day for five days for the management of moderate to severe URTIs in a real-life scenario.

## Materials and methods

Study design and duration

This study involved a subgroup analysis of moderate to severe category of patients with URTIs from a multicenter, retrospective, observational, real-world evidence study conducted across 184 ENT centers in India over 12 weeks [8].

Patient characteristics

Medical records of adult patients (aged over 18 years) with upper URTIs, who had provided prior consent for the use of their data in future research, were reviewed. These patients had been treated with azithromycin 500 mg once daily for five days, in accordance with local prescribing guidelines, and had completed follow-up by day 5. Their data were retrieved, compiled, and analyzed. Subsequently, data of patients with moderate to severe URTIs were further analyzed.

Data retrieval method

Data matching the inclusion criteria was retrieved from patient medical records. Physicians extracted anonymized data at the source using specially designed case record forms (CRFs) (see Appendices). The focus was on three key signs, namely, pharyngeal erythema, tonsillar erythema, and exudates or plugs on the tonsils, as well as three key symptoms: sore throat or difficulty swallowing, fever, and interference with daily activities [9]. These signs and symptoms were scored on a 3-point scale (0-2), as outlined in Table 1.

**Table 1:** Clinical scoring system for URTIs URTIs: upper respiratory tract infections

Clinical parameters	Variables	Response
Clinical signs	Pharyngeal erythema/swelling	2: Severe
		1: Moderate
		0: Mild
	Tonsillar erythema/swelling	2: Severe
		1: Moderate
		0: Mild
	Presence of exudates/plugs in tonsils	2: Severe
		1: Moderate
		0: Mild
Clinical symptoms	Sore throat/swallowing pain	2: Difficult to swallow
		1: Sore but can swallow
		0: Mild sore throat
	Fever in °C	2: ≥38.6
		1: 37.6-38.5
		0: ≤37.5
	Interfering with daily activities	2: Absent from work
		1: Annoying but can work
		0: Very little

Safety data, including reported adverse events, were documented. The physician's clinical assessment of azithromycin's effectiveness was also recorded as the Clinical Global Impression (CGI) scale in the CRF, defined by improvement in the clinical scoring for the six previously described parameters as determined by the clinician.

Assessment

A score was calculated for each symptom and sign, along with a cumulative score encompassing all six parameters. Changes in scores on day 5 of azithromycin treatment were recorded [10,11]. The CGI scale of change was assessed on day 5 using a 7-point rating scale.

Outcome measures and statistical analysis 

Data management was conducted using Microsoft Excel (Microsoft Corporation, Redmond, Washington, United States). Categorical variables were presented as frequencies and percentages, while quantitative data were summarized as mean±standard deviation (SD). Treatment effectiveness was evaluated by applying a paired t-test to assess changes in mean scores from baseline to day 5 for the clinical parameters of URTIs. The McNemar-Bowker test was used to assess the proportion of patients showing improvement in symptom scores from baseline to day 5. A significance level of p<0.05 (95% confidence interval) was set to determine statistical significance.

Missing data handling

All collected data were reviewed for completeness and consistency prior to analysis. In cases where data were missing, the nature and extent of the missingness were assessed. If the proportion of missing data for any variable was less than 5%, a complete analysis was performed. No data were excluded solely due to missingness unless critical for primary outcome assessment.

Sample size

Convenience sampling was employed as a practical approach to collect data from patients meeting the inclusion criteria, utilizing available medical records from the 184 ENT centers across India within the specified time frame.

Ethical considerations

The study was conducted in accordance with Good Clinical Practice (GCP) standards. Data were retrieved from patients who had previously provided consent for the use of their information for future research purposes. All retrieved data underwent anonymization during the collection process to ensure confidentiality. Ethical approval for the study was obtained from the Indira IVF Hospital Institutional Ethics Committee in Mumbai (approval number: AP LtdA00002) and was registered with the Clinical Trials Registry-India (CTRI/2024/04/066497).

## Results

Baseline characteristics

Data of 884 patients were retrieved from the databases of 184 ENT centers across India. This subgroup analysis focuses on the data of 427 patients with moderate to severe URTIs who satisfied the inclusion criteria for assessment. The patient demographic parameters are mentioned in Table 2.

**Table 2:** Demographics and baseline parameters

Parameters	N	Male	Female	M/F ratio
Gender	408	225	183	1.2
Parameters	N	Mean±SD	Median (IQR)	Range
Age (years)	385	38.34±13.07	36(28,46)	18-81
Pulse rate (beats/min)	306	86.35±9.27	86(80,92)	60-114
Body weight (kg)	352	62.14±11.51	60.5(54,70)	40-99
Height (cm)	298	161.18±10.74	160(155,168)	123-188.98
Temperature (°C)	310	37.6±0.77	37.5(37,38.33)	35.56-39.5
Respiratory rate (per minute)	261	17.02±2.64	17(15,20)	12-22

Effectiveness outcomes

By day 5, the investigators reported a significant improvement in clinical scores across all six parameters (Table 3). From 40.7% of moderate to 59.3% of severe grade of pharyngeal erythema at baseline, only 16.6% patients had moderate erythema, and 0.2% patients had severe erythema at day 5. At baseline, 37% had moderate tonsillar erythema, and severe tonsillar erythema was observed in 63% of patients; however, by day 5, only 11.9% of patients had moderate erythema, and no cases of severe tonsillar erythema were noted. At baseline, 52.9% of patients were reported to have moderate and 47.1% of patients severe exudates on their tonsils, but by day 5, only 7.6% of patients continued to have moderate tonsillar exudates, while tonsillar exudates of the severe category were reported in only 0.2% of patients. At baseline, significant severity of sore throat was reported in 73.5% patients having difficulty in swallowing, and 26.5% patients had sore throat but could swallow. This decreased to 10.2% patients with sore throat but could swallow by day 5, and only 0.2% of patients reported having difficulty in swallowing. By day 5, fever resolution was seen in 96.7% of patients. Work absenteeism reduced significantly from 71.2% at baseline before treatment to 0.9% on day 5 after treatment with azithromycin (Table 4).

**Table 3:** Effectiveness outcomes: changes in mean clinical score # for mean scores: p-values calculated using paired t-test

Parameter	N	Baseline (day 1)	Day 5	Mean difference±SD	P-value#
		Mean±SD	Mean±SD		
Pharyngeal erythema/swelling	421	1.6±0.49	0.17±0.38	1.43±0.57	<0.001
Tonsillar erythema/swelling	421	1.64±0.48	0.12±0.32	1.52±0.55	<0.001
Presence of exudates/plugs in tonsils	420	1.48±0.5	0.08±0.28	1.4±0.53	<0.001
Sore throat/swallowing pain	422	1.74±0.44	0.11±0.32	1.63±0.5	<0.001
Fever in °C	419	1.56±0.5	0.03±0.18	1.53±0.51	<0.001
Interfering with daily activities	422	1.72±0.45	0.09±0.32	1.63±0.51	<0.001
Total score	427	9.7±2.02	0.59±1.24	9.1±2.23	<0.001

**Table 4:** Effectiveness outcomes: proportion of patients showing improvement in symptom score # for % scores: p-values calculated using the McNemar-Bowker test

Clinical parameter	Severity	N	Baseline %	N	Day 5 (%)	P-value# (change from baseline)
							Pharyngeal erythema	Moderate	174	40.7%	70	16.6%	<0.001
Severe	253	59.3%	1	0.2%									
Tonsillar erythema	Moderate	158	37%	50	11.9%	N.A.
	Severe	269	63%	0	0%	
Presence of exudates/plugs in tonsils	Moderate	226	52.9%	32	7.6%	<0.001
	Severe	201	47.1%	1	0.2%	
Sore throat/swallowing pain	Sore but can swallow	113	26.5%	43	10.2%	<0.001
	Difficult to swallow	314	73.5%	1	0.2%	
Fever in °C	37.6-38.5	190	44.5%	14	3.3%	<0.001
	≥38.6	237	55.5%	0	0%	
Interfering with daily activities	Annoying but can work	123	28.8%	31	7.3%	<0.001
	Absent from work	304	71.2%	4	0.9%	

As per the investigator's rating for 384 patients, 95.6% of patients had much to very much improvement in the CGI scale with azithromycin in the treatment of URTIs (Table 5).

**Table 5:** Investigator-reported Clinical Global Impression scale at day 5

Clinical Global Impression scale	Number of patients assessed	Percentage of patients assessed
Very much improved	195	50.8%
Much improved	172	44.8%
Minimally improved	16	4.1%
No change	0	0%
Minimally worse	1	0.3%
Much worse	0	0%
Total	384	100%

Safety assessment

Among the 427 patients, nine (2.1%) reported adverse events during the course of the treatment with azithromycin, indicating a favorable safety profile of azithromycin in the treatment group. The majority of adverse events were mild in severity and gastrointestinal that were reported in eight patients.

## Discussion

Tonsillopharyngitis is part of the spectrum of URTIs. Patients with GABHS pharyngitis typically exhibit a sudden onset of fever, severe throat pain, difficulty swallowing, an inflamed pharynx, enlarged and erythematous tonsils, a red and swollen uvula, and tender anterior cervical lymph nodes [12]. Prompt diagnosis and antimicrobial treatment are crucial to prevent complications, reduce symptom severity, and decrease disease transmission [13]. The duration of antibiotic treatment significantly impacts patient adherence and overall healthcare costs. Shorter treatment regimens, such as a five-day course of azithromycin, are preferred to improve compliance and reduce the likelihood of bacterial resistance [14]. Five-day course of azithromycin is often considered important to ensure adequate pathogen clearance, reduce the risk of treatment failure, and potentially lower the emergence of antimicrobial resistance that may arise from subtherapeutic exposure associated with shorter regimens [15]. A recent systematic review has concluded that azithromycin exhibited superior efficacy, with a favorable safety profile, and fewer adverse effects in treating URTIs [16]. A meta-analysis highlighted that a shorter duration of azithromycin is also effective, further enhancing patient adherence and minimizing the risk of developing bacterial resistance [17].

The results from this study indicate that azithromycin 500 mg once daily for five days significantly improves clinical outcomes in patients with moderate to severe acute URTIs. Significant reduction in scores underscores the effectiveness of azithromycin in alleviating pharyngeal erythema, tonsillar erythema, tonsillar exudates, sore throat, and fever, associated with tonsillopharyngitis. In this study, azithromycin administration was linked to a marked decrease in work absenteeism among patients with moderate to severe tonsillopharyngitis. By the fifth day of treatment, both the symptoms and clinical signs of tonsillopharyngitis were fully resolved. In the current study, only nine (2.1%) patients reported adverse events throughout the treatment period. This shows that azithromycin was well-tolerated across the study population. These findings highlight the favorable safety profile of azithromycin in the given clinical setting, supporting its use as a well-tolerated therapeutic option.

The CGI scale on day 5 strongly suggests that the treatment was highly effective for the vast majority of patients, with over 99% (383 out of 384) showing some level of improvement, out of which 96% showed either very much or much improvement and 43 patients were not assessed by the clinician. The data also indicates that the treatment was effective, with very few patients experiencing no change or worsening conditions. This further supports the effectiveness and safety of the treatment regimen, providing compelling evidence for its use in clinical practice to manage the symptoms of moderate to severe URTIs. In the present study, by day 5, there was a notable decrease in symptom severity for key indicators such as pharyngeal and tonsillar erythema and tonsillar exudates as indicated by a reduction in mean score by 90%, 93%, and 95%, respectively. Also, significant improvement in sore throat and fever was reported as indicated by a reduction in mean score by 94% and 98%, respectively. This rapid symptom relief improves patient comfort and demonstrates the effectiveness of the treatment. These results support azithromycin as a preferred treatment option for managing moderate to severe URTIs, especially in cases where swift recovery is desired to reduce symptom burden and enhance patient quality of life. Furthermore, the low rate of adverse events makes azithromycin a favorable choice for patients, potentially increasing adherence to treatment and improving outcomes.

The rational use of antibiotics in URTIs is a critical aspect of clinical practice, particularly in the context of rising antimicrobial resistance. Most URTIs in adults are viral in origin and self-limiting, requiring only supportive care. Clinical guidelines emphasize that antibiotics should be reserved for cases with clear signs of bacterial infection, such as those caused by group A *Streptococcus*. The decision to initiate antibiotic therapy should be guided by clinical evaluation, symptom severity, and patient-specific factors, rather than routine or precautionary use. Promoting evidence-based prescribing practices helps preserve antibiotic efficacy, reduces the burden of resistance, and ensures optimal patient outcomes.

This study has limitations, including its retrospective nature, which introduces biases such as missing data, absence of a control drug, no microbiological confirmation through culture or rapid antigen detection test (RADT), recall bias, and the inability to analyze concomitant medications that may influence the overall course of treatment. The study's strength is rooted in its inclusion of a substantial real-world patient population and its multicenter design, which accurately reflects the continued sensitivity to azithromycin across various regions in India. This design also minimized location-specific biases, offering a more comprehensive view of azithromycin's effectiveness in treating URTIs nationwide. The clinical scoring system allowed for a standardized evaluation of symptoms, reducing subjective variability and increasing the reliability of the results. The rigorous methodology adopted in this research underpins the validity and quality of the findings.

## Conclusions

This multicenter Indian real-world study corroborates the effectiveness and safety of five-day azithromycin therapy in managing moderate to severe URTIs such as tonsillitis and pharyngitis, thereby reducing the chances of complications. The rapid symptom resolution leads to a reduction in interference with daily activities and early return to work in Indian patients with URTIs. Azithromycin is well-tolerated and is associated with a low incidence of adverse events. The high clinical scores assigned by the investigators reiterate the effectiveness and safety of azithromycin in treating moderate to severe URTIs in Indian patients. However, further randomized prospective studies are needed to validate and strengthen the outcomes observed in this study.

## Figures and Tables

**Figure 1:**
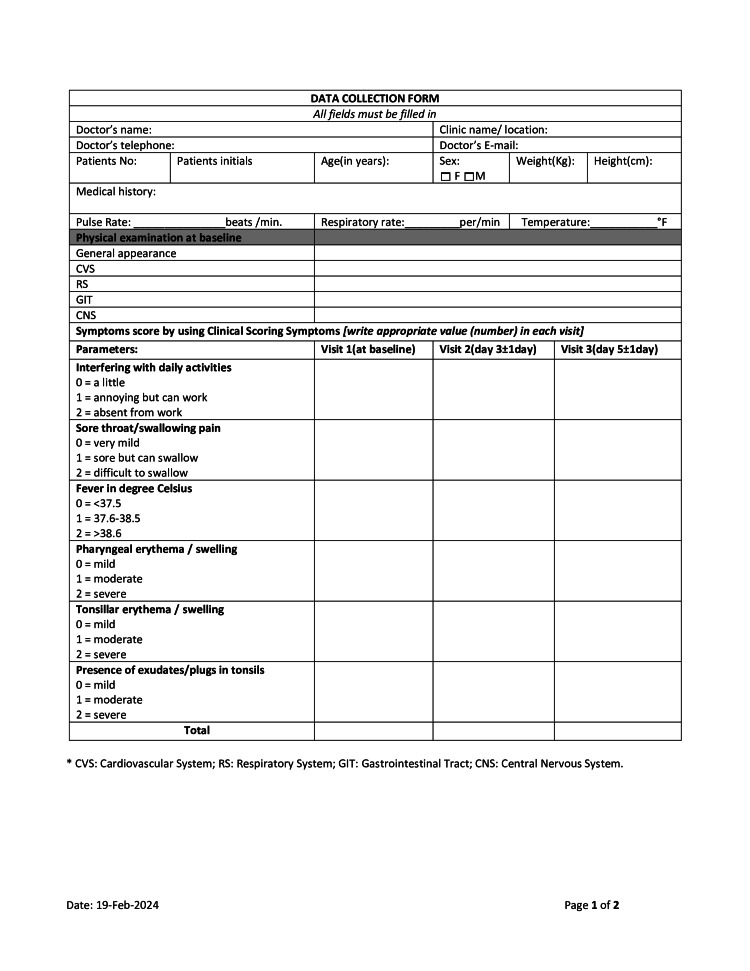
Case record form

**Figure 2:**
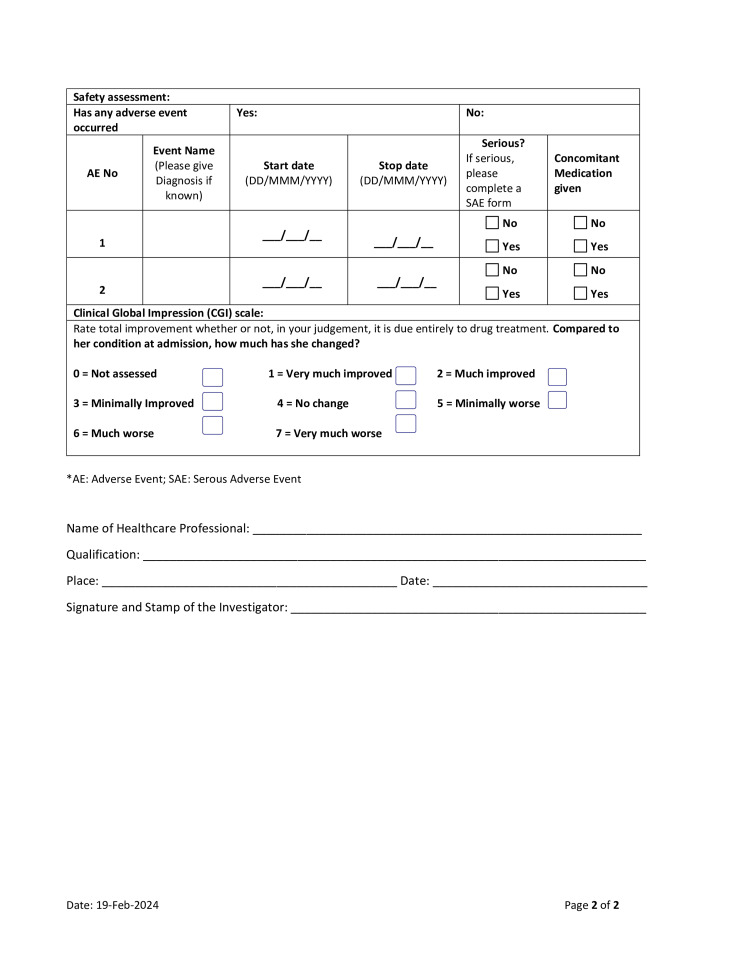
Case record form (continuation)
